# Initial Psychometric Evaluation of the Problematic Use of Generative Artificial Intelligence Scale Among Chinese School Students

**DOI:** 10.3390/bs16101763

**Published:** 2026-09-28

**Authors:** Yang Wang, Jiamin Huang, Deborah Baofeng Wang, Hongtao Shao, Xiaohan Liu, Tingjun Ye, Mei Peng, Hongsheng Yang, Yanqiu Yu, Joseph T. F. Lau

**Affiliations:** 1 The Affiliated Wenzhou Kangning Hospital of Wenzhou Medical University, Wenzhou 325000, China; 2 School of Mental Health, Wenzhou Medical University, Wenzhou 325035, China; 3 School of Public Health, Fudan University, Shanghai 200032, China; 4 Center for Health Behaviours Research, Jockey Club School of Public Health and Primary Care, The Chinese University of Hong Kong, Hong Kong SAR, China

**Keywords:** generative artificial intelligence (GAI), problematic use, Chinese school students, psychometric evaluation, mental health

## Abstract

Generative artificial intelligence (GAI) is increasingly used by school students, yet validated instruments for assessing problematic GAI use (PUGAI) in this population remain scarce. This study evaluated the psychometric properties of the Problematic Use of Generative Artificial Intelligence Scale (PUGAIS) in a large school-based sample of Chinese students. A cross-sectional survey included 19,484 students in Grades 4–9 who had used GAI. The sample was randomly split for exploratory and confirmatory factor analyses. Internal consistency, measurement invariance across sex, school type, and developmentally defined age groups, concurrent validity, latent classes, and a preliminary threshold were also examined. Following item refinement, the 8-item PUGAIS (PUGAIS-8) showed a dominant one-factor structure, although confirmatory model-fit evidence was mixed. The scale showed high internal consistency (Cronbach’s α = 0.940) and measurement invariance of item thresholds and factor loadings across sex, school type, and age groups. Ordinal sensitivity analyses indicated that the item-reduction results were partly sensitive to analytic treatment, although the PUGAIS-8 showed a more favorable overall structural fit than the original 10-item specification. A substantial floor effect indicated limited differentiation at the lower end of the construct. PUGAIS-8 scores were positively associated with depressive symptoms, anxiety symptoms, insomnia symptoms, and short-video addiction (r = 0.365–0.413, all *p* < 0.01). Latent class analysis identified a high-score subgroup (7.2%), and an exploratory ROC analysis yielded an internally derived descriptive threshold of 20.5. Overall, the findings provide initial psychometric support for the PUGAIS-8 as a brief measure of PUGAI among Chinese school students, while indicating the need for independent replication of the item-reduction findings and further evaluation of its measurement properties and proposed threshold.

## 1. Introduction

Generative artificial intelligence (GAI) has rapidly become integrated into how people search for information, solve problems, create content, and complete everyday tasks (Dwivedi et al., 2023; Xian et al., 2024). Advances in GAI have made increasingly sophisticated tools readily accessible to the public (Bommasani et al., 2021; Dwivedi et al., 2023), with particularly rapid uptake in educational contexts. A recent U.S. survey found that approximately 70% of adolescents had used at least one GAI tool (Common Sense Media, 2024), while studies in other educational settings have documented widespread use for homework, information seeking, creative activities, and research (Dorta-González et al., 2024; Miranda et al., 2024). This growing integration of GAI into learning and everyday life may be particularly relevant during adolescence, when learning habits, self-regulation, values, and mental health continue to develop (Lin, 2018).

GAI can support learning and information seeking, enhance creativity and efficiency, and assist with everyday decision-making (Hermann & Puntoni, 2024; S. Huang et al., 2024; Kolding et al., 2025). At the same time, concerns have been raised about excessive reliance on AI-generated information, reduced independent cognitive engagement, privacy and security, and unequal access to digital resources (S. Huang et al., 2024; Salah et al., 2024; Zhou & Zhang, 2024). These concerns may be particularly relevant to adolescents because critical thinking, autonomous decision-making, and self-regulation are still developing. Discussions of responsible AI use have therefore emphasized maintaining human oversight and avoiding uncritical dependence on AI systems (Thurzo, 2025). Beyond the frequency or intensity of GAI use, it is important to identify patterns involving impaired control, distress, or functional disruption. We refer to such patterns as problematic GAI use (PUGAI).

PUGAI may share features with other forms of problematic technology use, particularly impaired control and continued use despite negative consequences (Zhou & Zhang, 2024). Behavioral addictions have been conceptualized in terms of reinforcement processes and diminished behavioral control (Brand et al., 2019; Griffiths, 2005), while socially oriented digital activities may additionally be reinforced by interpersonal feedback and social rewards (Meshi et al., 2015). GAI differs in that much of its use is instrumental and goal-directed, including learning (Bai & Wang, 2025), information seeking (Lund et al., 2026), problem solving (Urban et al., 2024), and content generation (Conde et al., 2026). Frequent or prolonged GAI use may therefore reflect legitimate task demands rather than problematic involvement, and reliance on GAI may occur within reflective, cautious, and collaborative forms of problem solving (Hou et al., 2025a, 2025b). Moreover, impaired control and interpersonal consequences are conceptually distinguishable features of problematic technology use (Brand et al., 2019; Müller et al., 2022), suggesting that poorly controlled GAI use need not involve prominent interpersonal conflict. We therefore conceptualize PUGAI as poorly regulated GAI use accompanied by distress or functional consequences, rather than as an established behavioral addiction. Intensive or sustained use for legitimate academic or task-related purposes should not, by itself, be considered problematic solely because of its frequency or duration (Liao et al., 2026).

Empirical evidence on PUGAI among adolescents remains limited. One adolescent study reported prevalence estimates of 17.14% and 24.19% across two waves (S. Huang et al., 2024), although these estimates were based on a threshold that had not undergone formal psychometric validation. Other recent studies have adapted measures of problematic or addictive technology use to assess problematic GAI-related behaviors (Y. H. Chen, 2025; Y. Huang & Huang, 2025; S. C. Yu et al., 2024; Zhou & Zhang, 2024). However, some instruments have not undergone comprehensive psychometric evaluation; some focus on a single platform such as ChatGPT, and evidence of their applicability to adolescents remains limited. These measurement gaps complicate comparisons across studies and the accumulation of evidence regarding the prevalence, correlates, and developmental consequences of PUGAI. A psychometrically evaluated measure is therefore needed to distinguish intensive GAI engagement from patterns characterized by diminished control, discomfort, or functional impairment (Y. Chen et al., 2025), and to provide a consistent basis for examining antecedents, outcomes, and group differences.

Measurement approaches in adjacent areas provide a useful starting point. Emerging GAI studies have examined AI dependence among adolescents (S. Huang et al., 2024), problematic ChatGPT use (Y. H. Chen, 2025), and technology addiction to generative AI chatbots (Y. Huang & Huang, 2025), but these approaches differ in conceptualization, technological focus, and target population. In the broader problematic-technology literature, the Smartphone Addiction Scale—Short Version (SAS-SV; Kwon et al., 2013) assesses features such as impaired control, increasing involvement, discomfort when access is restricted, and functional consequences and has been psychometrically evaluated in adolescents. These features provide a useful measurement foundation, but GAI differs from smartphones and other digital technologies because its use is often instrumental and goal-directed (Dorta-González et al., 2024; Dwivedi et al., 2023; Miranda et al., 2024). Thus, although a measure of PUGAI may draw on established indicators of impaired control and functional consequences (Kwon et al., 2013; Zhou & Zhang, 2024), these indicators require empirical evaluation in the specific context of GAI.

Accordingly, the present study adapted items from the SAS-SV (Kwon et al., 2013) to assess PUGAI. The SAS-SV was selected not because GAI use was assumed to be equivalent to smartphone addiction, but because it captures general behavioral features potentially relevant to poorly controlled technology use, including difficulties regulating use, functional consequences, and discomfort when access is restricted. The resulting Problematic Use of Generative Artificial Intelligence Scale (PUGAIS) was intended as a brief measure of problematic patterns of GAI use rather than as a diagnostic instrument or a comprehensive measure of GAI-related harms.

The present study evaluated the psychometric properties of the PUGAIS in a large school-based sample of Chinese school students. We first examined its factor structure using exploratory and confirmatory factor analyses in independent split-half subsamples and assessed internal consistency and floor and ceiling effects. We then examined measurement invariance across sex, school type, and developmentally defined age groups and evaluated concurrent validity through associations with depressive symptoms, anxiety symptoms, insomnia symptoms, and short-video addiction. Finally, latent class analysis was used exploratorily to characterize heterogeneity in PUGAIS-8 item-response patterns, followed by receiver operating characteristic analysis to examine an internally derived preliminary threshold. Given the absence of an independent clinical criterion, this threshold was considered exploratory and was not intended to represent a diagnostic cutoff.

## 2. Materials and Methods

### 2.1. Participants and Procedure

A cross-sectional, school-based survey was conducted in March 2025 across 72 schools (51 primary schools and 21 secondary schools) in Wenzhou, Zhejiang Province, China. All students in Grades 4–9 at the participating schools were invited to participate. Participants had a mean age of 12.14 years (SD = 1.75; range = 7–17 years). The study was approved by the Medical Ethics Committee of the Affiliated Kangning Hospital of Wenzhou Medical University (approval No. YJ-2024-21-02).

Data were collected in classroom settings by trained fieldworkers using an electronic questionnaire system. Before questionnaire administration, students were informed of the study purpose, procedures, voluntary nature of participation, and their right to decline participation or withdraw at any time without penalty. The same information was provided on the questionnaire cover page. Parents or legal guardians were informed about the study in advance, and written informed consent was obtained before their children were invited to participate. Written informed assent was also obtained from participating students according to the ethics-approved procedure. Students whose parents or legal guardians declined participation were not invited to participate. No incentives were provided. Questionnaires were completed without teachers present and submitted directly through the survey system. Similar procedures have been used in previous school-based adolescent research (Y. Yu et al., 2024). The final dataset was de-identified before analysis to protect participants’ privacy and confidentiality.

A total of 40,120 questionnaires were returned. Of these, 19,165 (47.77%) were excluded because the respondents reported never having used GAI. Among the 20,955 students who reported prior GAI use, a further 1471 questionnaires were excluded because information on sex or grade was missing or because more than 20% of responses were missing across the key study measures, including the Problematic Use of Generative Artificial Intelligence Scale (PUGAIS), the 9-item Patient Health Questionnaire (PHQ-9), the 7-item Generalized Anxiety Disorder scale (GAD-7), the 7-item Insomnia Severity Index (ISI), and the 6-item short-video addiction scale. The final analytic sample comprised 19,484 students, representing 93.0% of those who reported prior GAI use.

Sociodemographic information included sex, school type (primary or secondary school), whether participants lived with both parents, parental educational attainment, self-reported academic performance (lowest 20%, 61–80% from the top, 41–60% from the top, 21–40% from the top, or highest 20%), self-reported household income (very low/low, average, high/very high, or not reported), and boarding-school attendance.

### 2.2. Measures

All study measures were administered in a single electronic survey session. The questionnaire consisted of sociodemographic questions followed by the study measures described below, with each measure presented as a separate section in a fixed order.

#### 2.2.1. Problematic Use of Generative Artificial Intelligence

The Problematic Use of Generative Artificial Intelligence Scale (PUGAIS) was adapted from the 10-item Smartphone Addiction Scale—Short Version (SAS-SV; Kwon et al., 2013). The Chinese SAS-SV has demonstrated good psychometric properties (Xiang et al., 2019) and has been widely used in Chinese samples (e.g., Lai et al., 2025; Xie et al., 2024). The SAS-SV was selected because it captures behavioral features relevant to poorly controlled technology use, including impaired control, functional impairment, and discomfort when access is restricted. PUGAI was not assumed to be equivalent to smartphone addiction.

For adaptation, smartphone-related wording was replaced with GAI-related wording, and several items were modified to fit students’ daily contexts. The translation and adaptation involved two behavioral scientists, two psychologists, one clinical psychologist, and one psychiatrist. DBW conducted the initial translation and adaptation, JTFL performed back-translation and further modification, and the research team reviewed the items through discussion, considering their relevance to PUGAI, comprehensibility for school students, and coverage of the intended construct. No formal content-validity assessment, cognitive interviewing, or separate pilot testing was conducted during the initial adaptation. The questionnaire defined GAI tools as systems capable of generating human-like content and provided examples of commonly available platforms in China.

Items were rated on a 4-point Likert scale (1 = strongly disagree to 4 = strongly agree), with higher scores indicating greater levels of PUGAI. No items were reverse-scored. The original 10-item version yielded scores ranging from 10 to 40. The original 10 items and the final retained items, with the original and final item numbers provided to show their correspondence, are presented in Supplementary S1.

#### 2.2.2. Depressive Symptoms

Depressive symptoms were assessed using the 9-item Patient Health Questionnaire (PHQ-9; Kroenke et al., 2001). The PHQ-9 has been validated in Chinese populations (Zhang et al., 2013) and has been used in previous studies in China (Guo et al., 2021). Participants rated the frequency of depressive symptoms during the preceding 2 weeks on a 4-point scale ranging from 0 (not at all) to 3 (nearly every day). Total scores range from 0 to 27, with higher scores indicating more severe depressive symptoms. In the present sample, the PHQ-9 showed excellent internal consistency (Cronbach’s α = 0.911).

#### 2.2.3. Anxiety Symptoms

Anxiety symptoms were assessed using the 7-item Generalized Anxiety Disorder scale (GAD-7; Löwe et al., 2008). The Chinese version of the GAD-7 has demonstrated good psychometric properties (Tong et al., 2016) and has been used in previous studies in China (Guo et al., 2021). Participants rated the frequency of anxiety symptoms during the preceding 2 weeks on a 4-point scale ranging from 0 (not at all) to 3 (nearly every day). Total scores range from 0 to 21, with higher scores indicating more severe anxiety symptoms. In the present sample, the GAD-7 showed excellent internal consistency (Cronbach’s α = 0.932).

#### 2.2.4. Insomnia Symptoms

Insomnia symptoms were assessed using the 7-item Insomnia Severity Index (ISI; Gagnon et al., 2013; Morin, 1993). The ISI assesses several aspects of insomnia, including difficulties with sleep initiation and maintenance, early morning awakening, satisfaction with current sleep patterns, interference with daily functioning, noticeability of sleep problems, and distress related to sleep difficulties. Items are rated on a 5-point scale ranging from 0 to 4, yielding a total score from 0 to 28, with higher scores indicating greater insomnia severity. In the present sample, the ISI showed good internal consistency (Cronbach’s α = 0.875).

#### 2.2.5. Short-Video Addiction

Short-video addiction was assessed using a 6-item measure adapted from the Bergen Social Media Addiction Scale (BSMAS; Andreassen et al., 2012). The items assess six behavioral features related to problematic short-video use and are rated on a 5-point Likert scale ranging from 1 (very rarely) to 5 (very often), with higher scores indicating greater levels of short-video addiction. The adapted measure has previously been used among Chinese school students and demonstrated acceptable reliability (Sun et al., 2024). In the present sample, the scale showed good internal consistency (Cronbach’s α = 0.874).

### 2.3. Statistical Analysis

A total of 92 item-level responses were missing across the 19,484 participants. Missing values were handled using multiple imputation (MI) with fully conditional specification (FCS), generating 20 imputed datasets (Graham, 2009). The imputation model included the PUGAIS items, depressive symptoms, anxiety symptoms, insomnia symptoms, short-video addiction, age, sex, and school type. The imputed data were used in subsequent psychometric analyses, with estimates combined across datasets where supported by the analytical procedure. Complete-case analyses were conducted as sensitivity analyses. Analyses were performed using IBM SPSS Statistics version 26.0, IBM SPSS Amos version 22.0, R version 4.4.1, and Mplus version 8.3. All statistical tests were two-tailed, with *p* < 0.05 considered statistically significant.

The psychometric evaluation was conducted sequentially. First, CFA using maximum likelihood estimation in IBM SPSS Amos version 22.0 was performed to evaluate the hypothesized one-factor structure of the original 10-item PUGAIS. Model fit was evaluated using χ^2^/df, CFI, TLI, RMSEA, and SRMR. Values of χ^2^/df < 5.0, CFI and TLI ≥ 0.90, and RMSEA and SRMR ≤ 0.08 were considered indicative of acceptable fit (Kline, 2015).

Because the initial 10-item model did not demonstrate satisfactory fit, the sample was randomly divided into an exploratory subsample (*n* = 9761) and a validation subsample (*n* = 9723) for scale refinement and cross-validation (Y. Yu et al., 2024). The primary item-reduction EFA was conducted in the exploratory subsample using principal axis factoring based on Pearson correlations with direct oblimin rotation. Items with primary factor loadings < 0.40 or substantial cross-loadings (≥0.40) were considered for removal (Field, 2013), with the solution re-estimated after each removal. The retained eight items were summed to calculate the PUGAIS-8 total score (range = 8–32). Parallel analysis based on polychoric correlations, using 1000 simulated datasets and the 95th percentile criterion, was used to further evaluate factor retention.

Given the four-category response format, ordinal sensitivity analyses were also conducted. The EFA was repeated using polychoric correlations, and the 10-, 9-, and 8-item specifications were evaluated using WLSMV with ordered categorical indicators. The models were compared in the same validation subsample to exclude differences in sample composition. These analyses complemented rather than replaced the primary item-reduction procedure. The final PUGAIS-8 was evaluated using WLSMV CFA in the independent validation subsample. School-level clustering was examined using ICCs and a cluster-adjusted CFA with robust maximum likelihood as a sensitivity analysis.

Internal consistency was evaluated using Cronbach’s α and ordinal McDonald’s ω, and inter-item correlations were examined for potential item redundancy. Floor and ceiling effects were defined as more than 15% of participants obtaining the minimum or maximum possible score, respectively (Terwee et al., 2007). Floor and ceiling effects were additionally examined across age groups, with differences evaluated using chi-square tests. Item-level response distributions were examined to characterize responses across the four categories. Concurrent validity was assessed using Pearson correlations of PUGAIS-8 scores with depressive symptoms, anxiety symptoms, insomnia symptoms, and short-video addiction.

Measurement invariance across sex, school type, and developmentally defined age groups was examined using multigroup CFA with WLSMV estimation and ordered categorical indicators (Putnick & Bornstein, 2016). Participants were grouped into three age categories (9–10, 11–12, and 13–15 years). Those aged 7–8 or 16–17 years were excluded from the age-group invariance analysis because of the small numbers at these age extremes (*n* = 35 in total) but remained included in the sex- and school-type analyses. Configural, threshold, and threshold-plus-loading invariance models were tested sequentially. Model fit was evaluated using χ^2^, CFI, TLI, RMSEA, and SRMR, with ΔCFI, ΔRMSEA, and ΔSRMR calculated between successive models. The prespecified primary criterion for measurement invariance was |ΔCFI| ≤ 0.01 (Cheung & Rensvold, 2002).

Latent class analysis (LCA) was conducted in Mplus version 8.3 as an exploratory person-centered analysis using the eight PUGAIS-8 items as categorical indicators. The items were modeled using the default categorical mixture-model parameterization, without additional within-class residual covariances. Models with two to five classes were estimated separately using 200 random sets of starting values, with 50 retained for final-stage optimization. Model selection considered AIC, BIC, likelihood-ratio tests, entropy, class size, substantive interpretability, and reproducibility across the exploratory and validation subsamples. Lower AIC and BIC indicated better relative fit, and entropy > 0.80 indicated good classification accuracy (Nylund-Gibson & Choi, 2018). Very small classes were interpreted cautiously. The LCA was used to characterize heterogeneity in PUGAIS-8 item-response patterns rather than to establish clinically or diagnostically distinct classes; the continuous PUGAIS-8 total score remained the primary representation of individual differences in PUGAI.

Finally, ROC analysis was conducted exploratorily to descriptively quantify the correspondence between the PUGAIS-8 total score and membership in the high-score class. Because both were derived from the same PUGAIS-8 item responses, the ROC analysis was not interpreted as evidence of criterion validity, diagnostic accuracy, or screening accuracy. The threshold was selected by maximizing the Youden index, with sensitivity and specificity also reported (Akobeng, 2007). The resulting threshold was considered internally derived and descriptive and requires validation against independent external criteria.

## 3. Results

### 3.1. Participant Characteristics

The final analytic sample comprised 19,484 participants, of whom 52.8% were boys and 49.5% were primary school students. Most participants (84.6%) lived with both parents, and 5.6% attended boarding schools. Regarding parental education, 36.5% of fathers and 35.0% of mothers had completed junior high school or below as their highest level of education. In addition, 40.9% of participants reported a high or very high household income. Detailed participant characteristics are presented in Table 1.

### 3.2. Psychometric Evaluation of the PUGAIS

#### 3.2.1. Factor Structure of the Original 10-Item PUGAIS

All standardized factor loadings for the original 10-item PUGAIS were statistically significant and ranged from 0.573 to 0.867 (all *p* < 0.001). However, the hypothesized one-factor model did not provide an acceptable overall fit to the data, χ^2^(35) = 11,944.535, χ^2^/df = 341.272, CFI = 0.920, TLI = 0.897, RMSEA = 0.132, and SRMR = 0.051. The factor structure was therefore further examined using EFA in an exploratory subsample and CFA in a separate validation subsample.

#### 3.2.2. Factor Structure of the PUGAIS-8

An EFA was conducted in the exploratory subsample to identify a more parsimonious factor structure, which was subsequently evaluated in the independent validation subsample.

In the exploratory subsample, sampling adequacy was excellent (KMO = 0.940), and Bartlett’s test of sphericity was significant, χ^2^(28) = 61,915.111, *p* < 0.001. Original Item 1 was removed because its loading was below the prespecified criterion of 0.40 (loading = 0.309). After re-estimation, Original Item 2 was also removed (loading = 0.396). The resulting PUGAIS-8 comprised Original Items 3–10, with loadings ranging from 0.583 to 0.772 (Table 2). The one-factor solution had an eigenvalue of 5.63 and explained 70.4% of the variance. Parallel analysis based on polychoric correlations also supported retention of a single dominant factor.

In the independent validation subsample (*n* = 9723), ordinal CFA using WLSMV yielded χ^2^(20) = 4453.77, CFI = 0.957, TLI = 0.940, RMSEA = 0.142, and SRMR = 0.027, with standardized loadings ranging from 0.811 to 0.929. Thus, although CFI, TLI, and SRMR were favorable, the elevated RMSEA indicated mixed evidence regarding the fit of a strictly unidimensional measurement model.

Sensitivity analyses further examined the effect of treating the four-category responses as ordinal. The polychoric EFA yielded higher loadings for Original Items 1 and 2; thus, their loading-based removal was not reproduced under the polychoric approach, indicating that the item-reduction results were sensitive to the correlation matrix used. In the identical complete-case validation subsample (*n* = 9723), WLSMV comparison of the 10-, 9-, and 8-item specifications showed a more favorable overall fit pattern for the PUGAIS-8 (CFI = 0.983, TLI = 0.976, RMSEA = 0.151, and SRMR = 0.027) than for the original 10-item model (CFI = 0.967, TLI = 0.958, RMSEA = 0.163, SRMR = 0.043). The maximum absolute residual correlation also decreased from 0.124 to 0.062. Detailed results of these ordinal sensitivity analyses are presented in Supplementary S2.

School-level clustering was modest (item ICCs = 0.013–0.024; total-score ICC = 0.023). In the cluster-adjusted sensitivity CFA, standardized loadings remained substantial (0.712–0.880), with CFI = 0.952, TLI = 0.933, RMSEA = 0.124, and SRMR = 0.032, indicating that accounting for school-level clustering did not materially alter the overall pattern of findings.

#### 3.2.3. Measurement Invariance Across Sex, School Type, and Age Groups

Measurement invariance results across sex, school type, and developmentally defined age groups are presented in Supplementary S3. For the age-group analyses, participants were categorized as 9–10 years (*n* = 2198), 11–12 years (*n* = 3379), and 13–15 years (*n* = 4111). Participants aged 7–8 or 16–17 years (*n* = 35 in total) were excluded from the age-group invariance analysis because their small numbers precluded the formation of adequately sized groups for multigroup analysis. The configural models showed CFI = 0.956, TLI = 0.938, RMSEA = 0.144, and SRMR = 0.028 across sex; CFI = 0.950, TLI = 0.930, RMSEA = 0.154, and SRMR = 0.028 across school type; and CFI = 0.950, TLI = 0.931, RMSEA = 0.154, and SRMR = 0.028 across age groups. Across the subsequent threshold and threshold-plus-loading models, CFI ranged from 0.950 to 0.956, TLI from 0.942 to 0.955, RMSEA from 0.145 to 0.179, and SRMR remained approximately 0.028. Changes in CFI between successive models were ≤0.001 and therefore below the prespecified criterion of |ΔCFI| ≤ 0.01. Based on the prespecified ΔCFI criterion, these results supported invariance of item thresholds and factor loadings across sex, school type, and the developmentally defined age groups. However, the consistently elevated RMSEA values indicate limitations in absolute model fit; therefore, the invariance findings should be interpreted cautiously. Full model-fit statistics, including χ^2^, degrees of freedom, and changes in RMSEA and SRMR, are reported in Supplementary S3.

#### 3.2.4. Internal Consistency and Floor and Ceiling Effects

The PUGAIS-8 demonstrated high internal consistency in the full analytic sample (*n* = 19,484; Cronbach’s α = 0.940; ordinal McDonald’s ω = 0.966), with similar estimates in the exploratory (*n* = 9761; α = 0.939; ω = 0.965) and validation subsamples (*n* = 9723; α = 0.940; ω = 0.965). In the full analytic sample, inter-item Pearson correlations ranged from 0.539 to 0.800, with the strongest correlation between Items 5 and 6 (*r* = 0.800), suggesting potential content overlap.

A substantial floor effect was observed, with 37.8% (*n* = 7363) of participants obtaining the minimum possible score, whereas the ceiling effect was negligible (0.9%, *n* = 170). Among participants aged 9–15 years, the proportion obtaining the minimum score ranged from 28.5% to 41.0% and exceeded the 15% criterion at every age. Floor-effect prevalence differed significantly across age groups, χ^2^(10) = 102.51, *p* < 0.001, although no clear monotonic age-related pattern was observed; ceiling-effect prevalence did not differ significantly, χ^2^(10) = 15.34, *p* = 0.120. Item-level response distributions are presented in Supplementary S4 and showed a consistent concentration in the lower response categories across all eight items, indicating that the floor effect was not driven by a single item. Complete-case sensitivity analyses yielded substantively unchanged conclusions regarding the main psychometric findings.

#### 3.2.5. Concurrent Validity

Concurrent validity was examined in the full analytic sample (*n* = 19,484). Individual PUGAIS-8 items were positively correlated with depressive symptoms (*r* = 0.304–0.361), anxiety symptoms (*r* = 0.293–0.357), insomnia symptoms (*r* = 0.300–0.336), and short-video addiction (*r* = 0.326–0.374; all *p* < 0.01).

At the scale level, higher PUGAIS-8 scores were positively associated with depressive symptoms (*r* = 0.396), anxiety symptoms (*r* = 0.387), insomnia symptoms (*r* = 0.365), and short-video addiction (*r* = 0.413; all *p* < 0.01). The complete correlation matrix is presented in Table 3.

### 3.3. Latent Class Analysis

Latent class analysis (LCA) was conducted in the full analytic sample (*n* = 19,484) using the eight PUGAIS-8 items as class indicators. Models with two to five classes were compared. The two-class solution yielded AIC = 275,084.803, BIC = 275,281.738, entropy = 0.974, and LMRT = 86,678.403 (*p* = 0.333). The three-class solution yielded AIC = 239,311.047, BIC = 239,578.877, entropy = 0.980, and LMRT = 35,393.610 (*p* < 0.001). The four-class solution yielded AIC = 230,910.461, BIC = 231,249.187, entropy = 0.988, and LMRT = 8324.938 (*p* = 0.228), whereas the five-class solution yielded AIC = 226,563.273, BIC = 226,872.895, entropy = 0.971, and LMRT = 4321.437 (*p* = 0.138). Although AIC and BIC continued to decrease, the LMRT supported the three-class over the two-class solution but did not support the addition of a fourth or fifth class. Moreover, the four- and five-class solutions each contained a very small class (1.60% and 1.63%, respectively), raising concerns about stability and substantive interpretability. A comparable three-class solution was identified in both the exploratory and validation subsamples (Supplementary S5). Considering the information criteria, likelihood-ratio tests, classification accuracy, class sizes, substantive interpretability, and reproducibility across subsamples, the three-class solution was retained.

The three classes comprised 55.2%, 37.5%, and 7.2% of the sample and were descriptively characterized as low-score, moderate-score, and high-score classes, respectively. These classes represent relative patterns of PUGAIS-8 item endorsement within the present sample and should be interpreted as exploratory, sample-specific groupings rather than as evidence that PUGAI is inherently categorical or that the classes represent independently validated clinical or diagnostic categories. Accordingly, the continuous PUGAIS-8 total score remained the primary representation of individual differences in PUGAI, with LCA serving as a complementary person-centered characterization of item-response heterogeneity (Figure 1).

### 3.4. Exploratory Internally Derived Threshold for the PUGAIS-8

As an exploratory analysis, ROC analysis was used to descriptively quantify the correspondence between the continuous PUGAIS-8 total score and membership in the high-score class, rather than to evaluate independent classification or diagnostic performance. The AUC was 0.998 (95% CI [0.997, 0.998], *p* < 0.001), and the maximum Youden index (0.929) corresponded to a PUGAIS-8 score of 20.5, with a sensitivity of 0.938 and specificity of 0.991 (Figure 2). However, because both class membership and the total score were derived from the same PUGAIS-8 item responses, these estimates reflect internal correspondence rather than classification performance against an independent external criterion. Accordingly, the threshold of 20.5 should be regarded only as an exploratory, internally derived descriptive threshold and not as evidence of diagnostic or screening accuracy.

## 4. Discussion

The present study evaluated the psychometric properties of the PUGAIS in a large school-based sample of 19,484 Chinese school students. The hypothesized one-factor model of the original 10-item version did not demonstrate adequate fit. Subsequent EFA in the exploratory subsample resulted in the removal of two items with factor loadings below the prespecified criterion, yielding the PUGAIS-8. In the independent validation subsample, the shortened scale showed favorable CFI, TLI, and SRMR values, although the elevated RMSEA indicated mixed evidence regarding strict unidimensionality. The PUGAIS-8 also demonstrated high internal consistency, measurement invariance of item thresholds and factor loadings across sex, school type, and the included age groups, and positive associations with depressive symptoms, anxiety symptoms, insomnia symptoms, and short-video addiction. Exploratory LCA further identified three score-based classes, and ROC analysis provided an internally derived descriptive threshold. Overall, these findings provide initial evidence regarding the internal structure, reliability, measurement invariance, and relations with external variables of the PUGAIS-8 rather than comprehensive evidence for the validity of the broader PUGAI construct.

The findings also help clarify the conceptualization of PUGAI. Although the PUGAIS was adapted from an established measure of problematic technology use, features derived from such measures may not transfer uniformly to GAI. GAI is often used instrumentally for learning, information seeking, problem solving, and content generation (Bai & Wang, 2025; Lund et al., 2026; Urban et al., 2024). Accordingly, frequent, prolonged, or increasing use may reflect legitimate educational or task-related demands rather than problematic involvement. Such use becomes more relevant to PUGAI when accompanied by impaired control, distress, or meaningful functional consequences. Similarly, reliance on GAI may take reflective, cautious, and collaborative forms without necessarily indicating impaired control (Hou et al., 2025b). This distinction is particularly important for interpreting the retained items assessing increased GAI use and highlights the need for further refinement of the operationalization of PUGAI.

The item-reduction findings nevertheless require cautious interpretation. Two items were removed during the primary scale-refinement procedure because their factor loadings were below the prespecified criterion of 0.40. However, polychoric sensitivity analysis yielded higher loadings for these items, indicating sensitivity to the treatment of ordinal responses. Ordinal CFA sensitivity analyses showed a more favorable overall fit for the PUGAIS-8 than for the original 10-item specification, with higher CFI and TLI, lower RMSEA and SRMR, and smaller residual misfit. This pattern remained when the models were estimated in the identical validation subsample. Substantively, the first removed item assessed continued GAI use despite negative interpersonal consequences, which may operate differently in GAI than in socially oriented digital activities reinforced by interpersonal feedback and social rewards (Meshi et al., 2015). The second assessed longer-than-intended use, which may reflect legitimate task demands rather than impaired control. Overall, the findings support retaining the more parsimonious PUGAIS-8 while indicating that the item-reduction results are partly sensitive to analytic treatment and require independent replication.

The measurement-model findings also warrant qualification. Based on the prespecified change-in-fit criterion, the PUGAIS-8 showed invariance of item thresholds and factor loadings across sex, school type, and the included age groups (9–10, 11–12, and 13–15 years), indicating similarity of these measurement parameters across groups under the specified model. However, the elevated RMSEA values across the baseline and subsequent invariance models warrant caution regarding absolute fit. Internal consistency was high across the full analytic sample and both subsamples according to Cronbach’s α and ordinal McDonald’s ω, but high internal consistency alone is insufficient evidence of measurement quality. In particular, the strong correlation between Items 5 and 6 (r = 0.800) may indicate content overlap or local dependence. Similarly, although parallel analysis supported a dominant factor, the elevated RMSEA in both the ordinal CFA (0.142) and cluster-adjusted CFA (0.124) indicated mixed evidence regarding a strictly unidimensional measurement model despite favorable CFI, TLI, and SRMR values. Possible explanations include local dependence, relatively homogeneous item content, and model misspecification, although these possibilities were not formally tested. Thus, the findings support a dominant factor but should not be interpreted as establishing strict unidimensionality. Future studies should examine local dependence, potential item redundancy, and alternative measurement models.

The substantial floor effect further limits the measurement properties of the PUGAIS-8 at the lower end of the construct. Item-level distributions showed consistently greater endorsement of the lower response categories, indicating that the floor effect was not driven by a single item and was also evident across the major age groups. This pattern may reflect genuinely low levels of PUGAI, limited item coverage or suboptimal response thresholds, or limited GAI engagement. Because detailed measures of GAI engagement were unavailable, these explanations could not be distinguished. Future studies should incorporate measures of GAI use frequency, duration, and intensity and examine item thresholds, discrimination, and measurement precision, including whether additional GAI-specific items could improve differentiation at the lower end of the construct.

PUGAIS-8 scores showed positive cross-sectional associations with depressive symptoms, anxiety symptoms, insomnia symptoms, and short-video addiction, broadly consistent with previous evidence linking problematic forms of digital technology use with psychological and behavioral correlates (Burkauskas et al., 2022; Sun et al., 2024). The moderate correlations indicate that PUGAIS-8 scores covaried with, while remaining distinguishable from, these constructs in the present sample. However, the cross-sectional design precludes conclusions regarding temporal ordering or causality. These findings provide preliminary evidence of theoretically expected associations within the nomological network of PUGAI, but broader construct validity requires additional evidence (Clark & Watson, 2019; Flake et al., 2017). The substantial intercorrelations among the mental health measures should also be considered, and these measures were not specifically selected to evaluate convergent or discriminant validity. An independent external criterion for criterion-related validity was also unavailable (Boateng et al., 2018). Future studies incorporating theoretically targeted comparison measures, independent indicators of functional impairment, and longitudinal data are needed to evaluate the convergent, discriminant, incremental, and criterion-related validity of the PUGAIS-8.

The exploratory LCA suggested heterogeneity in PUGAIS-8 item-response patterns, with 7.2% of participants classified into the high-score class. Because the classes were derived from the same item responses, they should be interpreted as exploratory, sample-specific groupings rather than as evidence that PUGAI is inherently categorical or as independently validated behavioral subtypes or risk groups. The comparable three-class solution observed across the exploratory and validation subsamples provides some evidence of stability within the present dataset, but independent replication and examination of external functional outcomes and longitudinal trajectories are needed.

Similarly, the ROC-derived threshold of 20.5 should be interpreted cautiously. Both the high-score class used as the reference and the PUGAIS-8 total score were derived from the same item responses; therefore, the very high AUC primarily reflects their internal correspondence rather than independent classification accuracy or criterion validity. The threshold should consequently be regarded only as exploratory, internally derived, and descriptive. Independent validation against external criteria, such as functional impairment or independently assessed difficulties in controlling GAI use, is required before considering its use for screening or clinical purposes. Neither the high-score class nor the threshold establishes a clinical diagnosis or a distinct behavioral addiction. Although the term generative artificial intelligence addiction syndrome has recently been proposed (Kooli et al., 2025), evidence regarding its clinical validity and diagnostic boundaries remains limited.

The findings have implications primarily for future research. The PUGAIS-8 provides a brief measure for studying problematic patterns of GAI use among school students, and its measurement invariance may facilitate comparisons across sex, school type, and the included age groups. However, its substantial floor effect limits fine-grained differentiation at lower scores. More broadly, the findings reinforce the distinction between problematic use and frequent, intensive, or increasing GAI engagement. Future school-based research could examine whether approaches promoting self-regulated and critically reflective GAI use are useful and whether students showing persistent control difficulties together with functional problems may benefit from more targeted support. These possibilities require prospective and intervention-based evaluation and should not currently be used to assign students to intervention levels or guide clinical decisions.

Several limitations should be considered. First, the absence of an independent clinical or functional criterion precluded external validation of the LCA-derived high-score class and ROC threshold. Second, the cross-sectional design precludes conclusions about temporal or causal relationships; longitudinal studies are needed. Third, the sample was recruited from Grades 4–9 in a single city in Zhejiang Province, China, limiting geographical, developmental, and cultural generalizability. Independent replication in more diverse samples is therefore required. Fourth, test–retest data were unavailable, precluding evaluation of temporal stability. Fifth, because the PUGAIS was adapted from the SAS-SV, it may not fully capture GAI-specific features; in particular, the retained increased-use items require further evaluation because increased use alone does not necessarily indicate PUGAI. Sixth, peer interaction was not assessed. Finally, the substantial floor effect and absence of detailed GAI engagement measures limited interpretation of lower scores. Future research using item response theory may help clarify item thresholds, discrimination, and measurement precision across the latent continuum.

## 5. Conclusions

The present study provides initial psychometric support for the PUGAIS-8 as a brief measure of PUGAI among Chinese school students. The findings support a dominant factor, although the elevated RMSEA indicates mixed evidence regarding a strictly unidimensional measurement model. The scale showed high internal consistency and measurement invariance across sex, school type, and the included age groups. Although the item-reduction results were partly sensitive to analytic treatment, sensitivity analyses showed more favorable overall structural fit for the PUGAIS-8 than for the original 10-item version. The substantial floor effect limits differentiation at lower scores, and the ROC-derived threshold should be considered preliminary and descriptive. Further independent, longitudinal, and criterion-based validation is needed to establish the validity, generalizability, and measurement precision of the PUGAIS-8.

## Figures and Tables

**Figure 1:**
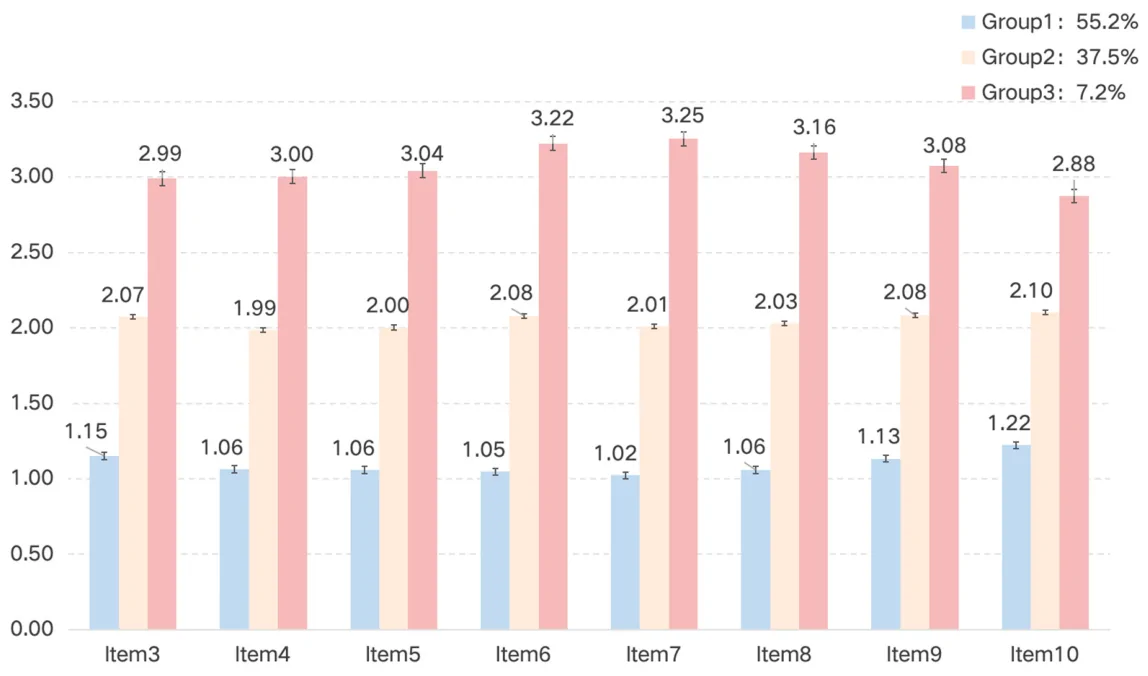
Latent class analysis results for the PUGAIS-8.

**Figure 2:**
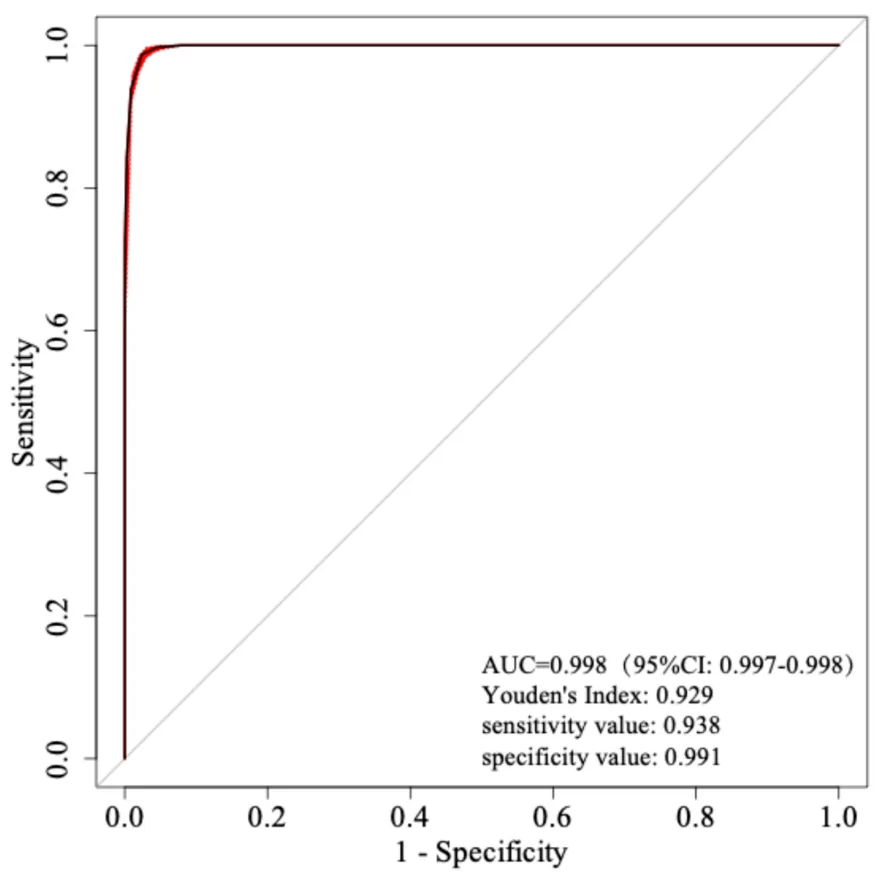
Exploratory receiver operating characteristic analysis of the PUGAIS-8 using the internally derived high-score class classification.

**Table 1:** Participant characteristics of Chinese school students who had ever used generative artificial intelligence (*n* = 19,484).

		*n*	%
**Sex**			
	Male	10,290	52.80%
	Female	9194	47.20%
**School type**			
	Primary school	9640	49.50%
	Secondary school	9844	50.50%
**Living with parents**			
	With both parents	16,427	84.60%
	With mother only	1239	6.40%
	With father only	637	3.30%
	Not living with any parents	1103	5.70%
**Father’s education level**			
	Junior high school or below	7088	36.50%
	Senior high school or equal	5411	27.80%
	College or above	5869	30.20%
**Mother’s education level**			
	Junior high school or below	6813	35.00%
	Senior high school or equal	5070	26.10%
	College or above	6512	33.50%
**Self-reported academic performance**			
	Lowest (Bottom 20%)	1298	6.70%
	Lower-middle (Bottom 21–40%)	3488	18.00%
	Middle (Middle 20%)	3973	20.40%
	Upper-middle (Top 21–40%)	5969	30.70%
	Top (Top 20%)	4701	24.20%
**Self-reported family income level**			
	Very low/low	1209	6.20%
	Average	9402	48.40%
	High/very high	7944	40.90%
	Not reported	868	4.50%
**Boarding school students**			
	Yes	1097	5.60%
	No	18,345	94.40%

**Table 2:** Original item numbers and exploratory and confirmatory factor loadings of the retained PUGAIS-8 items in the exploratory and validation subsamples.

Final PUGAIS-8 Items	Original PUGAIS Item	EFA (*n* = 9761) (Factor Loading)	CFA (*n* = 9723) (*β*)
Item 1. I have tried to spend less time using GAI tools, but I have been unable to do so.	Original Item 3	0.583	0.811
Item 2. I have experienced physical discomfort, such as sore eyes or muscle aches, due to prolonged use of GAI tools.	Original Item 4	0.659	0.858
Item 3. I habitually use GAI tools before going to sleep, which has reduced my sleep time or worsened my sleep quality.	Original Item 5	0.719	0.895
Item 4. My use of GAI tools has had negative effects on my academic or work performance.	Original Item 6	0.742	0.905
Item 5. I would feel distressed if I were suddenly restricted from using GAI tools.	Original Item 7	0.754	0.917
Item 6. I feel psychologically uncomfortable if I do not use GAI tools for a period of time.	Original Item 8	0.772	0.929
Item 7. I have noticed that the amount of time I spend using GAI tools has been increasing.	Original Item 9	0.749	0.906
Item 8. Compared with three months ago, the average amount of time I spend using GAI tools each week has increased substantially.	Original Item 10	0.656	0.861
Variance explained (%)		70.413%	

**Table 3:** Item-level and scale-level correlations of the PUGAIS-8 with external variables.

	Item 1	Item 2	Item 3	Item 4	Item 5	Item 6	Item 7	Item 8	PUGAI	Depressive Symptoms	Anxiety Symptoms	Insomnia Symptoms
**Item 1**	1											
**Item 2**	0.651 **	1										
**Item 3**	0.627 **	0.695 **	1									
**Item 4**	0.617 **	0.688 **	0.743 **	1								
**Item 5**	0.594 **	0.635 **	0.697 **	0.715 **	1							
**Item 6**	0.594 **	0.636 **	0.690 **	0.723 **	0.800 **	1						
**Item 7**	0.584 **	0.607 **	0.667 **	0.685 **	0.744 **	0.770 **	1					
**Item 8**	0.547 **	0.569 **	0.611 **	0.629 **	0.669 **	0.700 **	0.751 **	1				
**PUGAI**	0.778 **	0.817 **	0.850 **	0.860 **	0.869 **	0.877 **	0.862 **	0.815 **	1			
**Depressive symptoms**	0.304 **	0.332 **	0.321 **	0.320 **	0.361 **	0.360 **	0.337 **	0.327 **	0.396 **	1		
**Anxiety symptoms**	0.293 **	0.318 **	0.312 **	0.308 **	0.354 **	0.357 **	0.335 **	0.322 **	0.387 **	0.817 **	1	
**Insomnia symptoms**	0.300 **	0.315 **	0.315 **	0.310 **	0.331 **	0.336 **	0.321 **	0.303 **	0.365 **	0.711 **	0.656 **	1
**Short video addiction**	0.331 **	0.326 **	0.334 **	0.337 **	0.374 **	0.368 **	0.360 **	0.350 **	0.413 **	0.528 **	0.514 **	0.463 **

“**” indicates *p* < 0.01.

## Data Availability

Participant-level data are not publicly available because the study involved minors and data sharing is restricted by the applicable ethical and informed-consent requirements. Detailed information on scale scoring, statistical procedures, software, estimators, and model specifications is provided in the Methods and Supplementary Materials to facilitate reproducibility. Additional methodological information may be provided by the corresponding author upon reasonable request, where appropriate.

## Supplementary Materials

The following supporting information can be downloaded at: https://www.mdpi.com/article/10.3390/bs16101763/s1, Supplementary S1: 1. Chinese version of PUGAIS. 2. Chinese version of PUGAIS-8. Supplementary S2: Sensitivity comparison of the 10-item, 9-item, and 8-item PUGAIS specifications using WLSMV estimation in the identical validation subsample (*n* = 9723). Supplementary S3: Measurement invariance of the PUGAIS-8 across sex, school type, and age groups. Supplementary S4: Item-level response distributions of the PUGAIS-8 in the full analytic sample (*n* = 19,484). Supplementary S5: Cross-validation summary of model fit using the exploratory and validation subsamples: first subsample (*n* = 9761) and second subsample (*n* = 9723). Supplementary S6: Analytical Syntax.

## Abbreviations

The following abbreviations are used in this manuscript:

AIC	Akaike information criterion
AUC	Area under the curve
BIC	Bayesian information criterion
CFA	Confirmatory factor analysis
CFI	Comparative fit index
EFA	Exploratory factor analysis
GAD-7	7-item Generalized Anxiety Disorder scale
GAI	Generative artificial intelligence
ISI	Insomnia severity index
KMO	Kaiser–Meyer–Olkin measure of sampling adequacy
LCA	Latent class analysis
LMRT	Lo–Mendell–Rubin adjusted likelihood ratio test
MI	Multiple imputation
PHQ-9	9-item Patient Health Questionnaire
PUGAI	Problematic Use of Generative Artificial Intelligence
PUGAIS	Problematic Use of Generative Artificial Intelligence Scale
PUGAIS-8	8-item Problematic Use of Generative Artificial Intelligence Scale
ROC	Receiver operating characteristic
RMSEA	Root mean square error of approximation
SAS-SV	Smartphone Addiction Scale—Short Version
SRMR	Standardized root mean square residual
SVA	Short-video addiction
TLI	Tucker–Lewis Index
