# Supplementary material for: Initial Psychometric Evaluation of the Problematic Use of Generative Artificial Intelligence Scale Among Chinese School Students
Source: Behav Sci (Basel). 2026 Sep 28;16(10):1763. doi: 10.3390/bs16101763 (PMC13701801; doi:10.3390/bs16101763)
Supplement: Supplementary file 1 [file behavsci-16-01763-s001.zip › behavsci-4544203-supplementary.pdf]

Supplementary S1

1. Chinese version of PUGAIS

以下是关于生成式人工智能工具（如 DeepSeek 等）使用的一些描述，请根据你自己的一些情况，在相应的数字上打“√”。(The following statements describe the use of generative artificial intelligence (GAI) tools (e.g., DeepSeek). Please indicate the extent to which each statement applies to you by placing a “√” under the corresponding response option.)

		非常不同意 (Strongly Disagree)	不同意 (Disagree)	同意 (Agree)	非常同意 (Strongly Agree)
**1.**	**虽然使用生成式人工智能工具对我的日常人际关系造成了负面影响，我仍未减少使用 (Although using GAI tools has negatively affected my daily interpersonal relationships, I have not reduced my use.)**	1	2	3	4
**2.**	**使用生成式人工智能工具所花费的时间，常常超过自己本来预定的程度 (I often spend more time using generative artificial intelligence tools than I originally intended.)**	1	2	3	4
**3.**	我曾试过想花较少的时间在使用生成式人工智能工具上，但却无法做到 **(I have tried to spend less time using GAI tools, but I have been unable to do so.)**	1	2	3	4
**4.**	我曾因长时间使用生成式人工智能工具发生身体不适，如眼睛酸涩，肌肉酸痛 **(I have experienced physical discomfort, such as sore eyes or muscle aches, due to prolonged use of GAI tools.)**	1	2	3	4
**5.**	我习惯睡前使用生成式人工智能工具，因此减少睡眠时间或睡眠质量变差 **(I habitually use GAI tools before going to sleep, which has reduced my sleep time or worsened my sleep quality.)**	1	2	3	4

**6.**	使用生成式人工智能工具对我的学业或工作造成了一些负面影响<br>**(My use of GAI tools has had negative effects on my academic or work performance.)**	1	2	3	4
**7.**	如果突然限制我不能使用生成式人工智能工具，我会觉得很难受<br>**(I would feel distressed if I were suddenly restricted from using GAI tools.)**	1	2	3	4
**8.**	我只要有一段时间没有用生成式人工智能工具，就会觉得心里不舒服<br>**(I feel psychologically uncomfortable if I do not use GAI tools for a period of time.)**	1	2	3	4
**9.**	我发现自己使用生成式人工智能工具的时间越来越长<br>**(I have noticed that the amount of time I spend using GAI tools has been increasing.)**	1	2	3	4
**10.**	与三个月前比起来，平均而言，我每周使用生成式人工智能工具的时间比以前增加了许多<br>**(Compared with three months ago, the average amount of time I spend using GAI tools each week has increased substantially.)**	1	2	3	4

Note: The first two items highlighted in red were removed because they showed unsatisfactory performance in the exploratory factor analysis.

2. Chinese version of PUGAIS-8

以下是关于生成式人工智能工具（如 DeepSeek 等）使用的一些描述，请根据你自己的一些情况，在相应的数字上打“√”。(The following statements describe the use of generative artificial intelligence (GAI) tools (e.g., DeepSeek). Please indicate the extent to which each statement applies to you by placing a “√” under the corresponding response option.)

		非常不同意 (Strongly Disagree)	不同意 (Disagree)	同意 (Agree)	非常同意 (Strongly Agree)
**1.**	**我曾试过想花较少的时间在使用生成式人工智能工具上，但却无法做到 (I have tried to spend less time using GAI tools, but I have been unable to do so.)**	1	2	3	4
**2.**	**我曾因长时间使用生成式人工智能工具发生身体不适，如眼睛酸涩，肌肉酸痛 (I have experienced physical discomfort, such as sore eyes or muscle aches, due to prolonged use of GAI tools.)**	1	2	3	4
**3.**	**我习惯睡前使用生成式人工智能工具，因此减少睡眠时间或睡眠质量变差 (I habitually use GAI tools before going to sleep, which has reduced my sleep time or worsened my sleep quality.)**	1	2	3	4
**4.**	**使用生成式人工智能工具对我的学业或工作造成了一些负面影响 (My use of GAI tools has had negative effects on my academic or work performance.)**	1	2	3	4
**5.**	**如果突然限制我不能使用生成式人工智能工具，我会觉得很难受 (I would feel distressed if I were suddenly restricted from using GAI tools.)**	1	2	3	4
**6.**	**我只要有一段时间没有用生成式人工智能工具，就会觉得心里不舒服 (I feel psychologically uncomfortable if I do not use GAI tools for a period of time.)**	1	2	3	4
**7.**	**我发现自己使用生成式人工智能工具的时间越来越长 (I have noticed that the amount of time I spend using GAI tools has been increasing.)**	1	2	3	4

**8.**	**与三个月前比起来，平均而言，我每周使用生成式人工智能工具的时间比以前增加了许多**<br>**(Compared with three months ago, the average amount of time I spend using GAI tools each week has increased substantially.)**	1	2	3	4

Note: Original PUGAIS Items 1 and 2 were removed during scale refinement because their factor loadings were below the prespecified criterion. The retained Original Items 3–10 constitute the final PUGAIS-8 and were renumbered as PUGAIS-8 Items 1–8.

Supplementary S2 Sensitivity comparison of the 10-item, 9-item, and 8-item PUGAIS specifications using WLSMV estimation in the identical validation subsample (n = 9,723).

Model	χ^2	df	CFI	TLI	RMSEA (90% CI)	SRMR	Maximum absolute residual
PUGAIS-10	9002.44	35	0.967	0.958	0.163 [.160, .166]	0.043	0.124
PUGAIS-9	7327.78	27	0.973	0.964	0.167 [.164, .170]	0.037	0.119
PUGAIS-8	4453.77*	20	0.983	0.976	0.151 [.147, .154]	0.027	0.062

Note. All models were estimated using WLSMV with ordered categorical indicators in the identical complete-case validation subsample (n = 9,723). The models differed in the number of observed indicators and were therefore compared descriptively rather than using a nested-model χ^2 difference test. PUGAIS-9 excluded Original Item 1, whereas PUGAIS-8 excluded Original Items 1 and 2.

Supplementary S3 Measurement invariance of the PUGAIS-8 across sex, school type, and age groups.

Grouping variable	Model	χ^2	df	CFI	TLI	RMSEA	SRMR	ΔCFI	ΔRMSEA	ΔSRMR
Sex	Configural	4532.16	40	0.956	0.938	0.144	0.028	—	—	—
Threshold	3326.55	48	0.956	0.949	0.161	0.028	<.001		+0.017	
Threshold + loading	3118.61	55	0.956	0.955	0.145	0.028	<.001		−0.016	
School type	Configural	4773.75	40	0.95	0.93	0.154	0.028	—	—	—
Threshold	3669.29	48	0.951	0.942	0.172	0.028	<.001		+0.018	
Threshold + loading	3492.71	55	0.951	0.95	0.156	0.028	<.001		−0.016	
Age group	Configural	4700.38	60	0.95	0.931	0.154	0.028	—	—	—
Threshold	3548.38	76	0.951	0.946	0.179	0.028	0.001		+0.025	
Threshold + loading	3357.69	90	0.951	0.954	0.156	0.028	<.001		−0.023	

Note. ΔCFI, ΔRMSEA, and ΔSRMR represent changes relative to the immediately preceding invariance model. The absolute change in CFI ($|\Delta CFI| \leq 0.01$) was used as the prespecified primary criterion for evaluating invariance. Age groups were defined as 9–10 years, 11–12 years, and 13–15 years.

Supplementary S4 Item-level response distributions of the PUGAIS-8 in the full analytic sample (n = 19,484)

PUGAIS-8 item	Strongly disagree, n (%)	Disagree, n (%)	Agree, n (%)	Strongly agree, n (%)
Item 1 (Original Item 3)	9855 (50.6%)	6951 (35.7%)	1974 (10.1%)	704 (3.6%)
Item 2 (Original Item 4)	10506 (53.9%)	6353 (32.6%)	1962 (10.1%)	663 (3.4%)
Item 3 (Original Item 5)	11301 (58.0%)	6247 (32.1%)	1358 (7.0%)	578 (3.0%)
Item 4 (Original Item 6)	11123 (57.1%)	6453 (33.1%)	1349 (6.9%)	559 (2.9%)
Item 5 (Original Item 7)	10863 (55.8%)	6356 (32.6%)	1658 (8.5%)	607 (3.1%)
Item 6 (Original Item 8)	11181 (57.4%)	6381 (32.7%)	1372 (7.0%)	550 (2.8%)
Item 7 (Original Item 9)	10946 (56.2%)	6446 (33.1%)	1538 (7.9%)	554 (2.8%)
Item 8 (Original Item 10)	10437 (53.6%)	6444 (33.1%)	1991 (10.2%)	612 (3.1%)

Supplementary S5 Cross-validation summary of model fit using the exploratory and validation subsamples: first subsample (*n*=9761) and second subsample (*n*=9723)

No. of classes	Log-likelihood	*df*	AIC	BIC	ssaBIC	Entropy	*p*	Probability of classes (%)
First subsample (*n*=9761)								
2	-69452.668	25	138955.336	139134.892	139055.446	0.973	<0.001	57.03/42.97
3	-60361.573	34	120791.147	121035.343	120927.296	0.980	<0.001	55.51/36.97/7.52
4	-58316.092	43	116718.184	117027.021	116890.373	0.990	0.115	55.97/5.71/33.72/4.60
5	-57481.044	52	115066.088	115439.565	115274.317	0.960	0.442	49.27/6.92/3.04/33.92/6.84
Second subsample (*n*=9723)								
2	-63755.463	25	137560.926	137723.294	137643.853	0.976	<0.001	56.92/43.07
3	-59174.287	34	128416.573	128637.394	128529.354	0.982	<0.001	55.45/37.37/7.20
4	-58113.251	43	116312.502	116591.775	116455.137	0.990	0.241	4.21/32.89/53.37/9.53
5	-57108.429	52	114320.858	114658.585	114493.347	0.992	0.228	4.70/52.34/33.20/4.40/5.40

AIC: Akaike Information Criterion; BIC: Bayesian Information Criterion; ssaBIC: sample size adjusted BIC; *df*: number of parameters in the *k*-class model.

Supplementary S6 Analytical Syntax

1. Polychoric EFA and Parallel Analysis (R)

```
library(psych)

# Original 10-item PUGAIS
items10 <- data[, paste0("PUGAI", 1:10)]

# ------------------------------------------------------------
# Polychoric EFA sensitivity analysis
# ------------------------------------------------------------

poly10 <- psych::polychoric(items10)

efa_poly10 <- psych::fa(
  r = poly10$rho,
  nfactors = 1,
  n.obs = nrow(data),
  fm = "pa",
  rotate = "oblimin"
)

print(efa_poly10$loadings, cutoff = 0.20)


# ------------------------------------------------------------
# Final PUGAIS-8
# Original Items 3-10
# ------------------------------------------------------------

items8 <- data[, paste0("PUGAI", 3:10)]

poly8 <- psych::polychoric(items8)

efa_poly8 <- psych::fa(
  r = poly8$rho,
  nfactors = 1,
  n.obs = nrow(data),
  fm = "pa",
  rotate = "oblimin"
)
```

```
print(efa_poly8$loadings, cutoff = 0.20)


# ----------------------------------------------------------
# Parallel analysis based on polychoric correlations
# 1,000 simulated datasets; 95th percentile criterion
# ----------------------------------------------------------

set.seed(2026)

psych::fa.parallel(
  items8,
  fa = "fa",
  fm = "pa",
  cor = "poly",
  n.iter = 1000,
  quant = 0.95,
  plot = TRUE
)
```

2. Ordinal CFA Sensitivity Analyses (Mplus)

```
DATA:
  FILE = validation_complete_case.dat;

VARIABLE:
  NAMES =
    PUGAI1 PUGAI2 PUGAI3 PUGAI4 PUGAI5
    PUGAI6 PUGAI7 PUGAI8 PUGAI9 PUGAI10;

  USEVARIABLES =
    PUGAI1-PUGAI10;

  CATEGORICAL =
    PUGAI1-PUGAI10;

ANALYSIS:
  ESTIMATOR = WLSMV;

MODEL:
```

```
  PUGAI BY
    PUGAI1-PUGAI10;

OUTPUT:
  STANDARDIZED;
  RESIDUAL;
```

3. Latent Class Analysis (Mplus)

```
DATA:
  FILE = pugais_lca.dat;

VARIABLE:
  NAMES =
    PUGAI3 PUGAI4 PUGAI5 PUGAI6
    PUGAI7 PUGAI8 PUGAI9 PUGAI10;

  USEVARIABLES =
    PUGAI3-PUGAI10;

  CATEGORICAL =
    PUGAI3-PUGAI10;

  CLASSES = c(3);

ANALYSIS:
  TYPE = MIXTURE;
  STARTS = 200 50;

MODEL:
  %OVERALL%

OUTPUT:
  TECH1;
  TECH11;
  TECH14;

SAVEDATA:
  FILE = LCA_3class.dat;
  SAVE = CPROBABILITIES;
```
